# Uncertainty in the Tail of the Variant Creutzfeldt-Jakob Disease Epidemic in the UK

**DOI:** 10.1371/journal.pone.0015626

**Published:** 2010-12-23

**Authors:** Tini Garske, Azra C. Ghani

**Affiliations:** Department of Infectious Disease Epidemiology, Medical Research Council Centre of Outbreak Analysis and Modelling, Imperial College London, London, United Kingdom; Swansea University, United Kingdom

## Abstract

Despite low case numbers the variant Creutzfeldt-Jakob disease epidemic poses many challenges for public health planning due to remaining uncertainties in disease biology and transmission routes. We develop a stochastic model for variant CJD transmission, taking into account the known transmission routes (food and red-cell transfusion) to assess the remaining uncertainty in the epidemic. We use Bayesian methods to obtain scenarios consistent with current data. Our results show a potentially long but uncertain tail in the epidemic, with a peak annual incidence of around 11 cases, but the 95% credibility interval between 1 and 65 cases. These cases are predicted to be due to past food-borne transmissions occurring in previously mostly unaffected genotypes and to transmissions via blood transfusion in all genotypes. However, we also show that the latter are unlikely to be identifiable as transfusion-associated cases by case-linking. Regardless of the numbers of future cases, even in the absence of any further control measures, we do not find any self-sustaining epidemics.

## Introduction

The incidence of variant Creutzfeldt-Jakob disease (vCJD) in the UK has declined considerably since the epidemic peaked in 2000, with currently less than 5 cases arising each year [1]. However, 15 years after the identification of the first vCJD case [2], there are still huge uncertainties governing many aspects of the epidemiology. Exposure via the primary route of infection – namely BSE-infected cattle entering the human food supply – remains at very low levels given the declining BSE epidemic in cattle and the remaining controls in place [3]. However, there remains concern about the possibility of future cases arising both from past exposure in previously unaffected genotypes and through person-to-person transmission. The latter is warranted given that 3 of the 171 cases due to definite, probable or possible vCJD to the end of 2009 have been linked to blood transfusions [4], [5], [6] and hence these could herald the start of a potential secondary wave of cases of unknown scale. Furthermore, it is thought that surgery and dentistry could potentially also harbour a risk of vCJD transmission as current decontamination methods might not be sufficient to remove infectivity from surgical and dental instruments [7], [8]. However, to date there is no evidence that any transmissions via these transmission routes have actually occurred.

The scale of any future waves depends in part on the existing prevalence of infection in the population. There is currently no simple diagnostic test for infection that can provide unambiguous estimates of prevalence. However, there have been several studies which have provided estimates of the prevalence of vCJD infection in appendix and tonsil tissues. In risk assessments, a positive tissue sample is taken as equivalent to that person being infectious [9], [10], [11], [12]. However, while this is the prudent approach, it is not clear how these tests correlate with infectiousness. Furthermore, it is still not known when in the incubation period detectable levels of PrPSc, the abnormal form of the prion protein, begin to accumulate in different tissues.

The prevalence of infection found in the British population [13], [14] is much higher than would be expected from the case data alone, indicating the existence of a subclinical carrier state, during which individuals may or may not be infectious, but will never develop clinical disease [15]. If these sub-clinically infected people are indeed infectious, they might play an important role in onward transmission via blood transfusion. However, as they may never present as cases, any transmissions caused by a sub-clinically infected donor cannot be linked to the blood-borne transmission route. It is therefore possible that some of the past cases that are currently attributed to food-borne transmission were indeed caused by human-to-human transmission.

The genotype of codon 129 of the Prion protein appears to be important for transmission dynamics, with the two alleles Methonine (M) and Valine (V) occurring in the population. All probable and definite cases to date that were genotyped were homozygous for MM, a genotype shared by about 40% of the British population. However, in 2009 a possible case was identified in a person of MV genotype [16]. Unfortunately, no post-mortem was performed on this person, precluding definite diagnosis. One explanation for the observed excess of cases in individuals with the MM genotype is that those with MV and VV genotypes are substantially less susceptible to vCJD disease than MMs. Under this scenario we would not expect to see many cases in these genotypes in the future. However, other hypotheses are conceivable, including very long incubation periods in non-MM genotypes, potentially resulting in a substantial number of cases arising in the future [17].

These large uncertainties in the tail of the epidemic pose a problem for public health planning as the expected magnitude of the future epidemic will have implications for the effort and resources needed to control it. Here we develop a stochastic model for vCJD spread via primary transmission through the consumption of BSE contaminated beef and secondary human-to-human transmission through red cell transfusions (but ignoring other blood components and transmissions via surgery or dentistry), taking into account the genetic structure of the population at codon 129 of the PrP protein. We fit the model to the available epidemiological data accounting for unobserved infections (pre-clinical and sub-clinical) and unobserved transmission via red cell transfusion to obtain the range of future scenarios consistent with the observed epidemic to date in order to quantify the uncertainty in the future risk of vCJD transmission in the UK and provide appropriate inputs for public health planning.

## Methods

### Data

**Figure 1:**
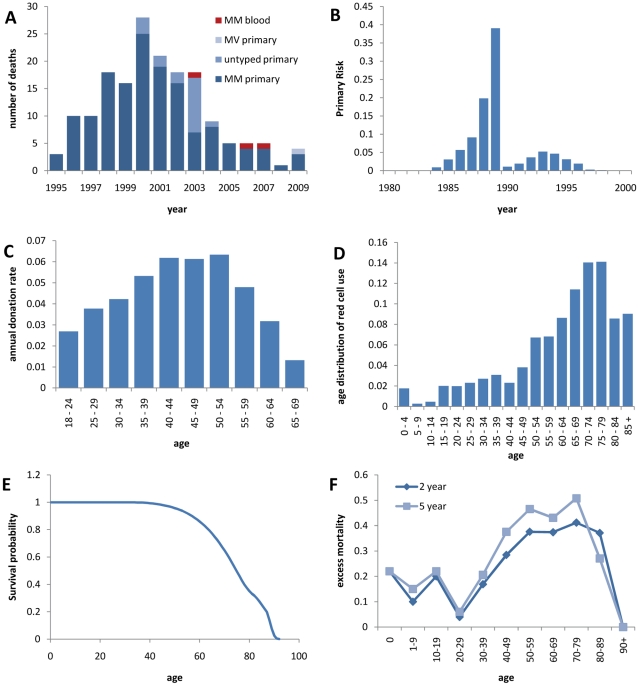
Input data. A Time series of observed cases by genotype and presumed transmission route, B time-dependence of the exposure of the British population to the BSE agent, C age-dependent annual blood donation rates, D age-distribution of red cell use, E Age-dependent survival probability, F age-dependent excess mortality associated with red cell transfusions.

To the end of 2009 there were 167 deaths from probable or definite vCJD in the UK, 3 of which are attributed to blood (red-cell) transfusions, and a further 4 deaths due to possible vCJD (see Figure 1A for the time-series, [18] for details on the classification of cases). 153 of these cases were genotyped, with 152 MMs and 1 MV (the MV case was classified as a possible case).

The prevalence of vCJD in the British population has been investigated by testing appendix [14] and tonsil [13] samples. Hilton et al. found 3 positives in 11,246 appendix samples collected between 1995 and 2000, the majority of which (91%) came from the highest risk birth cohorts 1961–1985 (Table 1). As a follow up to this study, the National Anonymous Tonsil Archive was set up, aiming to collect 100,000 pairs of tonsils from 2004 onwards. No positive samples have been found through testing with dual enzyme immune assay in the 85,000 samples tested by March 2010. However, in a sub-sample of 10,000 tissues tested using immuo-histochemistry, one follicle in a single sample tested positive using two different antibodies. Here we use the prevalence data from the original appendix study, but exclude the data from the NATA study for the model fitting as the most precautionary figure for risk management purposes as advised by the Spongiform Encephalopathy Advisory Committee [19].

**Table 1:** Details of the batches in the prevalence study by Hilton et al. [14].

Birth cohort	Number of samples	Number positive
1941–1960	574	0
1961–1985	10278	3
1986–1990	394	0

All tissues were removed between 1995 and 2000.

In the framework of the TMER (Transfusion Medicine Epidemiology Review) study [20] the cohort of patients who have received blood transfusions from donors that later developed vCJD is tracked. To date, 3 cases were attributed to transmission through blood transfusions, as the patients had received a red cell unit from a donor who later developed clinical disease. A further infection is thought to have occurred in a person of MV genotype who died 5 years after the transfusion in question without developing any symptoms of vCJD, but whose spleen tested positive in the post-mortem [21]. Two further vCJD cases potentially share a blood donor, who remains alive 20 years after the first donation but could nevertheless be the transmission source for both cases. However, due to incomplete medical records this cannot be confirmed, and taking this into account it is also possible that this link between the two cases is a chance event [22]. This potential link between these cases highlights that if indeed the proportion of subclinical infections is high, as the prevalence data suggest, and sub-clinically infected people are as infectious as those with pre-clinical infection, we would be able to identify only a small proportion of blood transfusion transmissions, as any transmission from donor to recipient where donor or recipient are sub-clinically infected cannot be detected. This means that theoretically, cases that are currently being attributed to primary transmission could potentially have been caused by blood transfusions.

While the fate of the whole cohort who have received transfusions from known pre-clinical donors (duration of survival post transfusion, and the development or not of clinical symptoms) sheds light on the transmissibility of vCJD via blood transfusions, the success of the transmission process depends on several parameters, such as the timing of the onset of infectivity in the donor, the level of infectivity, the susceptibility of the recipient and the duration of the incubation period in the recipient, which are difficult to disentangle with the numbers of transfusions implicated. Therefore we do not use these data to restrict the input ranges of our parameters, but rather fit our model to the 3 cases identified to have been caused by blood transfusions.

In order to reduce the risk of transmission via red cell transfusion, leuko-depletion of red cell units, i.e. removal of white blood cells, was introduced in 1998. This is thought to reduce the infectivity of blood by approximately 40% [23], however, as a single red cell unit may contain several thousand infectious doses, this reduction of infectivity might not have any impact at all on transmission. Following the identification of the first red cell transfusion associated case in 2003, since 2004 all recipients of blood transfusions have been excluded from donating blood. If this donor ban is effective and blood transfusions are the only viable human-to-human transmission route, this should break the cycle of onward transmissions and only allow a first generation of blood transfusion transmissions to occur. Both of these interventions are included in our model.

The dietary exposure to the BSE agent is estimated to have peaked in 1989, after which the specified bovine offal ban was introduced therefore reducing the number of infectious cattle entering the human food chain despite the continuing rise of the BSE epidemic itself, which peaked in 1993, see Figure 1B and [24].

Interestingly, the age distributions of red cell donors and recipients differ substantially: While the peak age for donations is between the early 40 s and the mid 50 s, with people over 70 years of age excluded from donating blood, the majority of red cells is used in the elderly, with a peak in the 70 s (see Figure 1C and D and [10]). These data, including the annual number of transfusions and the average number of red cell units given in a single transfusion dependent on the recipient's age, are taken from [25].

In our model, we assume a constant population size of 60 million with demographics that are stable over time. Data on the age structure of the UK population are taken from UK census data [10] (Figure 1E). Approximately 39% of the population are of MM genotype, 50% of MV and 11% of VV genotype [26].

We calculate the age dependent excess mortality associated with red cell transfusions (Figure 1F) from the probability of surviving until 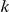 years post-transfusion 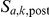 [25] and the survival data for the general population 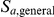 as 
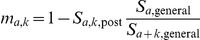
, where 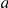 indexes age. As the majority of the excess mortality happens shortly after the transfusion, we use the 2-year post transfusion excess mortality and implement this excess mortality to occur at the time of the transfusion, therefore effectively preventing clinical disease and further onward transmission from any person not surviving the transfusion.

### Mathematical Model

We developed a stochastic simulation model for the transmission of vCJD in the British population which includes as transmission routes primary transmission through consumption of BSE contaminated beef as well as secondary or human-to-human transmission via red cell transfusions. This model is similar in structure to the deterministic model used in [10]. However, here we structure the population not only with respect to age (allowing for yearly age groups), but also to genotype, taking into account the 3 different genotypes at PRP codon 129, MM, MV and VV. Furthermore, in the stochastic individual-based framework we can keep track of the infection history and therefore classify the arising secondary cases into identifiable or unidentifiable with respect to the transmission route, depending on whether the blood donor in question did or did not develop clinical vCJD.

**Figure 2:**
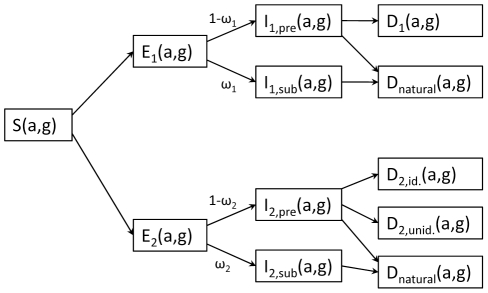
Flowchart of the model. The population is stratified by age (a) and genotype (g). S =  susceptible, E_1/2_ =  exposed via primary/secondary infection, I_1/2,pre_ =  preclinically infectious for primary/secondary infection, I_1/2, sub_ =  subclinically infectious for primary/secondary infection, D_1_ =  death from primary vCJD disease, D_natural_ =  death from other causes than vCJD, D_2,id._ =  death from identifiable transfusion associated vCJD disease, D_2,unid_ =  death from transfusion transmitted vCJD disease, but transmission route unidentifiable, ω_1_ =  proportion of subclinical infection for primary transmission, ω_2_ =  proportion of subclinical infection for transfusion associated transmission.

The natural history of the disease is modelled as a SEIR-type model, see Figure 2, which differentiates between primary (food-borne) and secondary (blood-borne) transmission. Furthermore, infection can lead to clinical disease after a lengthy incubation period, or be sub-clinical, in which case the infected person will continue to live to the end of their natural life span without any symptoms. The proportion 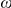 of infections that are sub-clinical varies between primary and secondary transmission. Upon infection, a person enters a non-infectious latent stage, followed by an infectious stage and eventually, death from either vCJD or natural causes (also allowing for age-dependent competing risks of death for pre-clinical infection).

For pre-clinical infection, the durations of the latent and infectious stages are sampled from gamma distributions for each individual, resulting in a gamma distributed total incubation period from infection to death from vCJD. Gamma distributions are chosen for ease of modelling as they only have two parameters and convenient summation properties, yet show a sufficient flexibility in shape to warrant a good model fit.

We parametrise the gamma distribution of the total incubation period by the mean 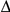 and shape parameter 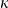 and specify the proportion 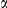 of the incubation period that should be infectious. From this we then find the mean and shape parameters of the gamma distributions for the latent and infectious stages as 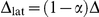, 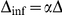, 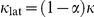 and 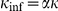. For sub-clinical infection, the latent stage is assumed to follow the same distribution as that of pre-clinical infection, but the infectious stage lasts until the end of the natural life span.

The population is stratified into 3 different genotypes, and here we allow the mean incubation period, but not the shape parameter, to vary between the different genotypes. Furthermore, the mean incubation period for secondary (blood-borne) transmission deviates from that for primary transmission by a factor 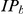, but again retaining the same shape parameter, such that the mean incubation period for genotype 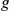 with secondary infection is 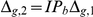, where 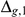 is the mean incubation period for genotype 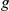 with primary infection.

For primary transmission, we fit a strong age-dependence of susceptibility, 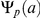, which could be caused by biological or by dietary factors or a combination of both [27], [28]. This is again modelled to follow a gamma distribution, yielding a good model fit to the data. For secondary transmission, we assume no age dependence in biological susceptibility, such that the age profile of infections is entirely determined by the age distribution of transfusion recipients. We allow different susceptibilities 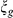 for the different genotypes (setting 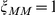). In the absence of compelling data suggesting otherwise, we assume that the genotype dependence of susceptibility is independent of transmission route. An alternative assumption that does not introduce yet more unknown parameters into the model would be to assume that all genotypes are equally susceptible to secondary transmission, as was done by [10], essentially ignoring the genetic structure of the population.

### Primary Infection

The risk of primary infection varies by age, genotype and calendar time via exposure to BSE-infected animals entering the food supply. The infection rate from primary transmission 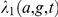 for a person of genotype 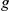, aged 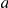 at time 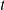 is given by
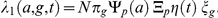



Here, 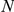 is the total UK population size and 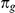 is the proportion of the population of genotype 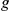. 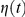 is the time-dependent exposure of the population to the BSE agent and 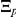 is the overall level of infectivity of BSE via the dietary route, normalised to the expected number of clinical cases arising from primary transmission in the MM genotype group, including past and future cases. A proportion 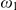 of infections are sub-clinical and the remaining proportion 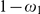 of infections are pre-clinical.

### Person-to-person transmission via red cell transfusion

People receive red cell transfusions according to a Poisson process with age-dependent rates. The number of units they receive also depends on their age. The probability that a patient of age 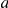 and genotype 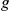
becomes infected given that they receive a red cell transfusion is given by
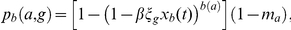
where 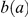 is the mean number of red cell units given in a transfusion to a person of age 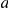, 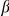 is the transmissibility, 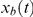 is the prevalence of infectivity in the blood pool and 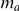 is the age-dependent excess mortality associated with the transfusion, assuming as a reasonable approximation that transfusion-associated excess mortality occurs close to the time of transfusion.

The rate of infection from red cell transfusions for a person of age 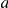 and genotype 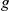 is then given by 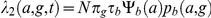
with 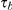 the annual number of red cell transfusions per person. For red cell transfusion-associated transmissions, the proportion of subclinical infections is denoted by 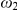.

Individuals are assumed to donate blood at random time points according to a Poisson process, where the donation rate only depends on the age of the donor. The prevalence of infectivity 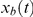 in the national blood pool at any time is assumed to equal that in the donor population (i.e. the prevalence in the whole population weighted by the age-dependent donation rates), 
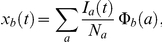
where 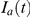 is the number of infectious individuals of age 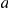 at time 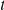, 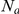 is the population size of age 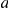, and 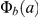 is the age-dependent donation rate.

The model is implemented as a branching process in continuous time, neglecting the depletion of susceptibles given that the overall prevalence of infection is estimated to be low. The infection events from primary and secondary infection follow Poisson processes with time-dependent rates 
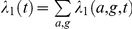
 and 
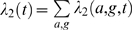
, such that the time of the next infection event is drawn from a Poisson distribution with rate 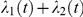. At each infection event, the transmission route and age and genotype of the infected person are determined according to the relative contributions of the individual rates. The latent and incubation periods are drawn from the respective gamma-distributions and the prevalence of infection in the population and in the blood pool over time is updated. Depending on the transmission route, and whether the case develops clinical disease, the count of deaths is updated at the appropriate time in the future.

### Model Parameterisation

Although we use all available data to parameterise the model, there are still many model parameters that are inherently unknown, mostly relating to the disease biology. In the model fitting process, we sample these from reasonable ranges, in order to assess which ranges of model parameters produce simulations consistent with the observed outbreak and to gain insight into the potential scale of the future epidemic. A summary of these unknown parameters and the ranges considered here is given in Table 2. For the parameters which were estimable the ranges were chosen such that they well covered the region containing the bulk of the posterior distributions, or cut off at values beyond which the dynamics of the model would not be altered. For instance, the range for the incubation period shape parameter was cut off at 100. A shape parameter this high corresponds to a highly peaked distribution, and any higher shape parameter would not influence the model output. Most of the non-estimable parameters have a theoretically possible range that is restricted to the interval 0–1, which was fully explored. Only the multiplier for the incubation period of secondary transmission could theoretically take values higher than the limit of 2 we have used here. However, any higher value would mean that the incubation period for blood-borne transmission is more than twice as long as that of primary food-borne transmission, which appears unlikely.

**Table 2:** Model parameters, prior distributions (all uniform within the range quoted) and medians (95% credibility intervals) of the fitted parameter values for those parameters that were estimable.

Description	Name	Prior distribution	Posterior median (95% credible interval)
Transmissibility via primary transmission	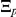	U[80, 230]	173 (144–199)
Mean incubation period for MMs infected via primary transmission	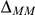	U[10,13.5]	11.6 (10.9–12.2)
Incubation period shape parameter	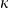	U[5, 100]	45 (23–81)
Mean age-dependent susceptibility/exposure for infection via primary transmission	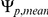	U[10, 40]	17.9 (15.9–20.0)
Age-dependent susceptibility/exposure shape	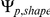	U[1], [15]	3.8 (2.3–6.6)
Mean incubation period for MVs infected via primary transmission	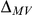	U [10, 80]	34 (19–73)
Mean incubation period for VVs infected via primary transmission	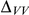	U[10, 80]	52 (26–77)
Relative susceptibility of MVs compared to MMs	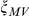	U[0, 1]	NE
Relative susceptibility of VVs compared to MMs	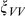	U[0, 1]	NE
Transmissibility via red cell transfusions	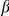	U[0, 1]	NE
Ratio of incubation period for red cell transfusion-associated transmissions compared to incubation period for primary transmission	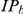	U[0, 2]	NE
Proportion of the incubation period that individuals are infectious	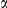	U[0, 1]	NE
Proportion of primary infections that are subclinical	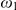	U[0, 1]	NE
Proportion of secondary infections that are subclinical	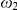	U[0, 1]	NE
Basic reproduction number	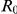	n/a	0.0056 (0.0003–0.0146)

NE  =  not estimable.

As a baseline scenario we assume that leuko-depletion results in a 40% reduction in the transmissibility 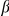 from 1998 onwards. We assume that the donor ban is only 90% effective. Both sensitivity and specificity of the test used in the appendix study are assumed to be 100%. Sensitivity analyses to these assumptions can be found in Supplementary Material S1.

While the time-dependence of the dietary exposure to BSE 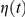 can be obtained from data on the BSE epidemic, the overall level of infectivity 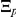 can only be gleaned from the number of primary human cases that have occurred to date; this parameter is varied in the model simulations. Furthermore, the relative susceptibility of the different genotypes 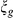 is unknown. We normalise the susceptibility of the MM genotype to 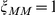, and vary the susceptibilities of the other genotypes between 0 and 1.

In order to reproduce the observed age-profile of cases we use an age-dependent susceptibility to primary infection 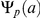, which we assume to be gamma-distributed, with mean 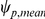 and shape parameter 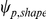, both of which are varied in the fitting process.

The transmissibility via red cell transfusions 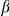, the proportion of the incubation period that is infective, 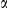, as well as the proportion of infections that are sub-clinical for primary and secondary transmission, 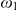 and 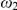, respectively, are unknown and varied across their possible range from 0 to 1.

### Model fitting

To fit the model to the time-series of the observed epidemic, including all 171 deaths from definite, probable and possible vCJD observed to the end of 2009 as well as the prevalence estimates from the appendix study [14], we use a Bayesian framework [29]. As there is little external information on which to base priors, we assume uniform (non-informative) priors for all our parameters. We therefore proceed by sampling the parameter space of the unknown parameters using Latin Hypercube sampling [30]. For each set of parameter values, we perform 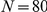 stochastic simulations and for each simulation calculate the likelihood of this parameter set based on these simulations. We weight each parameter set by its likelihood across the 80 stochastic realisations to obtain the posterior distribution for the parameters and for the outputs of interest (time-series of cases by transmission route).

The likelihood factorises into a factor for the time-series and its age distribution, a factor for the number of identified blood cases, a factor for the genotype distribution and a factor for the prevalence,
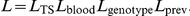



For the time-series likelihood 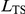, we stratify the observed incidence into 3 age groups, the cut-off points of 26 and 33 years roughly corresponding to the tertiles of the observed age-at-death distribution. We then group the incidence into year-groups, such that the number of cases 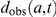 observed in each age-group 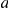 and year-group 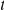 is at least 5. The full time-series likelihood is given by the product over all compartments,
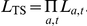



For each compartment we assume that both observed and simulated deaths accumulate according to a Poisson process with rate 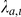. From the 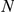 simulation runs per parameter set we infer the maximum likelihood estimate of this rate as 
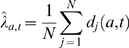
. The likelihood factor for this age- and year-group is then given by 
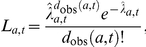



Similarly, we assume that the number of transfusion associated cases accumulates according to a Poisson process with rate 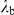, with maximum likelihood estimate 
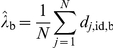
, where 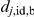 is the number of identifiable blood cases in simulation run 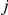. We therefore have 
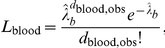
with the number of observed transfusion associated cases 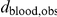.

We assume that the number of cases in the different genotypes arise according to a multinomial distribution. We infer the proportion 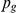 of cases of genotype 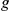 based on the 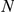 simulation runs per parameter set via maximum likelihood as
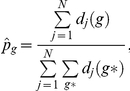
where 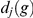 is the number of deaths in genotype 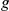 and simulation run 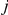, and the possible genotypes 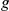 are MM, MV and VV. The genotype likelihood 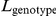 is then given as 
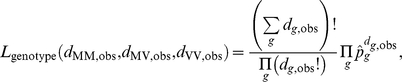



From the prevalence study using appendix tissues [14], we have information on 3 batches of tested tissue samples, which were collected between 1995 and 2000. For each batch 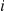, we know the range of birth cohorts, the time-span over which samples were collected, the total number 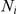 of samples and the number 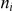 of positive samples, see Table 1. For each batch, we calculate the prevalence of infectiousness (assuming that being infectious coincides with testing positive) in the relevant age-range and time-span across all simulation runs as 
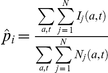
, where 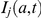 is the number of infectious people in the relevant age and year group, and 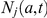 is the population size in the age- and year group in simulation run 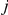. The probability of observing the number of positives observed in batch 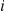, given the simulated prevalence 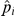 in the tested sub-population, is that of a binomial distribution, 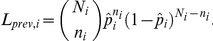



The overall prevalence likelihood is the product over all batches, 
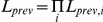
.

The results shown here are based on 1592100 sets of parameter values, sampled uniformly from the parameter ranges given in Table 2.

## Results

**Figure 3:**
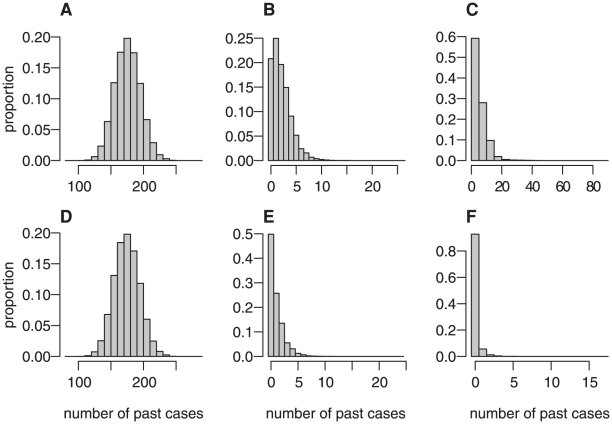
Posterior distribution of simulated case numbers to the end of 2009. A all cases, B identifiable blood cases, C unidentifiable blood cases, D MM cases, E MV cases, F VV cases.

A good fit to the total number of past cases, the number of past identifiable red cell transfusion associated cases and the distribution of cases to date by genotype was obtained (Figure 3). The total number of past cases obtained in the simulations is clustered around the observed value of 171 cases. The vast majority of these simulated cases are in the MM genotype, with small numbers of cases in the MV or VV genotype, matching the observed number of cases in these genotypes (1 and 0, respectively). At present, 3 cases have been associated with red-cell transfusion as the likely route of infection, well within the range recovered in the simulations. Of note, the number of past cases that are attributed to unidentifiable red-cell transfusion associated transmission (if one of either the donor or recipient has not yet developed symptoms) in the simulations is relatively small (less than 20 in the vast majority of scenarios). This suggests that to date primary transmission via consumption of BSE-infected cattle is most likely the main transmission route.

**Figure 4:**
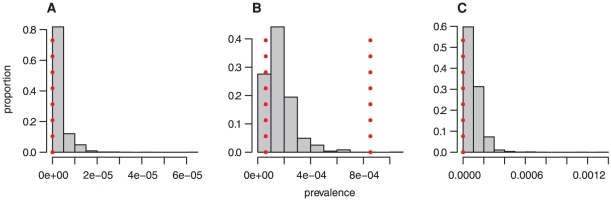
Posterior distribution of the prevalence of detectable PrPSc by batch as defined in the survey [**14**]. A 1941–1960 cohort, B 1961–1985 cohort, C 1986–1990 cohort. The vertical dotted lines indicate exact binomial confidence intervals for the prevalence based on the number of samples tested and the number of positives. For A and C, the upper end of the confidence interval lies to the right of the scale due to the smaller number of samples in these batches.

Figure 4 shows the range of the simulated prevalence in the age groups tested in the appendix study [14]. This falls for the most part within the 95% confidence bounds from this study. However, the simulated prevalence tends to be towards the lower end of the 95% confidence intervals estimated in the appendix survey.

Estimates for the model parameters are shown in Table 2, alongside the range explored for each parameter. Parameters determining primary transmission in the MM genotype (the group in which the vast majority of cases were observed) are well characterised and can be estimated with a reasonable degree of precision. The model parameters relating to red cell transfusion associated transmission as well as those relating to transmission in other genotypes cannot be estimated at present due to the small number of cases observed for these transmission routes and genotypes. Thus we can only vary them within reasonable ranges to get likely scales of the total outbreak size.

The estimated mean incubation period (defined here as the time from infection to death) for infection via primary transmission in the MM genotype is similar to previous estimates obtained from primary transmission models [28], [31] and is approximately the delay from the peak of exposure to BSE which occurred in 1989/1990 to the peak in the vCJD deaths which occurred in 2000. Age-dependent susceptibility/exposure is estimated to be highest in teenagers and young adults in line with previous estimates [27], [28].

**Figure 5:**
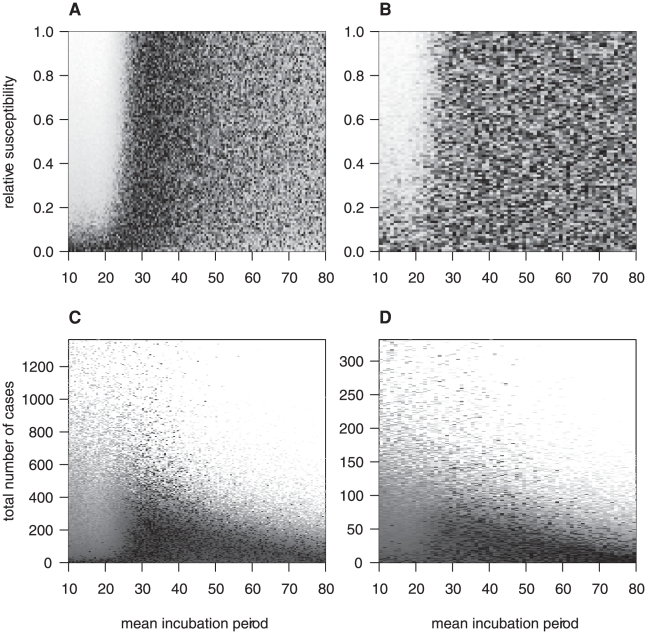
Joint posterior density of relative susceptibility and total number of cases with mean incubation period. A and B: relative susceptibility vs mean incubation period, C and D: total number of cases vs mean incubation period. A and C for MV genotypes, B and D for VV genotypes. Dark  =  high density, light  =  low density.

Whilst only one possible vCJD case has been observed in a non-MM genotype, this information does give some bounds on the mean incubation periods in these other genotypes in conjunction with their relative susceptibility compared to the MM-genotypes (Figure 5). The lack of cases in the VV genotype implies that short incubation periods in this genotype (i.e. comparable to that for the MM genotype) are only consistent with the observed epidemic if the susceptibility is highly suppressed. This is similar to the situation in the MV genotype. However, here very long mean incubation periods are also unlikely due to the recent possible case. Note that for longer incubation periods, the projected number of clinical cases is limited by the competing risk of death from other causes.

Table 2 also shows values of the basic reproduction number 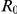, calculated based on the values of the input parameters for every simulation run (see Supplementary Material S1). 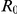 can be interpreted as the average number of secondary infections a typical infected case will generate during their infectious period in the absence of any saturation effects, and is therefore a threshold quantity: For values smaller than 1, each new generation of cases will be smaller than the previous generation, and the epidemic will die out with certainty even without any further control measures. However, there might be a substantial number of cases before this extinction occurs, particularly for values of 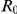 only a little less than 1. For values larger than 1 on the other hand, every subsequent generation of cases is on average larger than the previous, leading to exponential growth and a self-sustaining epidemic unless stochastic extinction occurs early on. For any of the scenarios that fit the observed epidemic, the basic reproduction number, calculated in the presence of the control measures of leuko-depletion and the donor ban, is much lower than this threshold, therefore precluding the potential for a self-sustaining epidemic and limiting the number of future cases substantially. The reason that our projections of future cases might appear large given the small values of 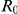 is that through the food borne transmission route, a large initial number of infectives (including the sub-clinical carriers) have been introduced into the population.

**Figure 6:**
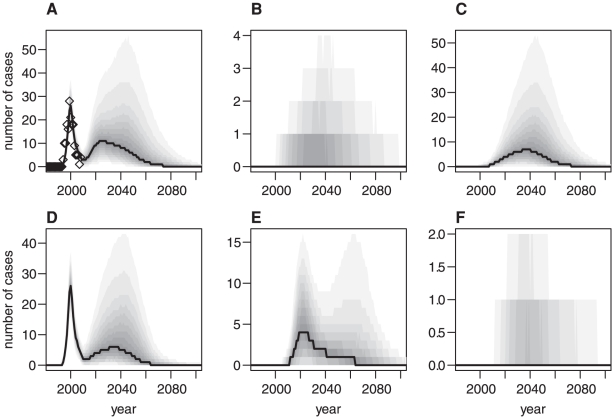
Median and posterior distributions of projected time series. A Total number of cases, B transfusion associated cases that can be identified through donor-recipient pairing, C unidentifiable transfusion associated cases and D to F number of cases in the different genotypes, MM, MV and VV, respectively. Diamonds  =  observed epidemic, solid line  =  median, greyscale graduations: 10% range to 90% range.

Figure 6 shows the posterior distribution of the time series of cases in total and stratified by transmission route as well as genotype whilst the median and 95% credible intervals for the cumulative number of future cases by genotype are given in Table 3 (note however that these cases are predicted to occur over the next hundred years). The projected time series of cases shows a double-peaked pattern. The first peak, which occurs around the year 2000, describes the past epidemic attributable mostly to exposure via primary transmission in the MM genotype and has a reasonably tight credibility range as this is fitted to data. Panel A also shows the observed number of cases, which agree well with the model fit of the time series. The second peak is broader, lasting from now (2010) to around 2080, with a peak value reached between 2020 and 2035, depending on the relative contribution of secondary cases in the MM genotype and primary cases in the MV genotype, with a most likely peak incidence of approximately 10 cases per year. However it should be borne in mind that the associated uncertainty for these projections is large, with the 95% credibility interval of the posterior distribution of the projected peak annual incidence ranging from 1 to 65 cases. While it therefore remains entirely possible that there will not be any observed secondary epidemic, the possibility of a secondary peak cannot be discounted despite the current low level of incidence.

**Table 3:** Estimated medians (95% credibility intervals) of the cumulative number of future cases from 2010 to 2179 by genotype and transmission route.

	Transmission route			
Genotype	Total	Primary	Identifiable blood	Unidentifiable blood
All genotypes	390 (84–3000)	100 (11–220)	17 (1–220)	260 (30–2700)
MM	200 (20–2200)	1 (0–6)	12 (0–160)	190 (16–2000)
MV	160 (4–980)	91 (1–210)	4 (0–57)	51 (1–760)
VV	13 (0–85)	7 (0–36)	0 (0–5)	5 (0–51)

Numbers are rounded to two significant digits.

If the secondary peak does occur, our projections suggest that it will mostly consist of transfusion-transmitted cases, mainly in the MM genotype, but to a lesser extent also in the MV genotype. However, our projections also suggest that we are unlikely to identify this as the route of transmission via linking of donor-recipient pairs due to the presence of subclinical infections and competing risks of death in the older group of transfusion recipients. Furthermore, there might also be a number of primary cases in the MV genotype, whereas the total number of cases in the VV genotype is expected to be low. While for the future primary cases generally transmission already has occurred, for the bulk of the transfusion associated peak the transmission is predicted to happen in the future (see Supplementary Material S1). These cases would therefore be preventable if appropriate and effective control measures, such as a blood test for vCJD or an effective method to remove infectivity from any blood products, were available.

It might be surprising at first to see that the number of projected future cases in the MV genotype is of the same order of magnitude as that in the MM genotype, whereas in the past, the vast majority of cases were observed in the MM genotype, with only one possible MV case. However, comparing the projected total numbers of future cases in the different genotypes is slightly misleading: In the MM genotype the primary epidemic is essentially over, whereas if any substantial number of primary MV cases occurs, this will be in the future due to a longer incubation period in this genotype. Therefore we should rather compare the projected numbers of transfusion-associated cases in the MM and MV genotypes, and here the MV cases make only about a third to a quarter of the MM cases, and the lower bound is close to 0, indicating that the possibly most intuitive scenario with very few future cases in the MV genotype is perfectly consistent with the model projections. However, due to uncertainties in parameters such as the incubation period, we cannot exclude a considerably larger epidemic in this genotype either. The same argument of course applies also to VV cases, but due to the small proportion of the VV genotype in the population, the numbers involved are much smaller than for the MV genotype.

## Discussion

When the first vCJD cases were reported in the late 1990s, the small numbers combined with lack of knowledge of both the potential transmission routes and key epidemiological parameters meant that projections of the future epidemic were highly uncertain [24], [32]. Following the peak in cases in 2000, and their subsequent decline to low numbers, it has for several years been possible to characterise the oral transmission route in the MM genotype and estimate associated epidemiological parameters with a reasonable degree of precision [28], [31]. However, with small numbers of cases now arising in different genotypes and via other transmission routes (3 cases of MM genotype attributed to blood transfusions since 2003 and reports of a possible vCJD case in a person of MV genotype), there remain concerns about the potential for a second epidemic wave.

Building on previous work [10], [17], we have used a stochastic model in a Bayesian framework, combining the transmission via food-borne and red cell transfusion associated transmission with a differentiation with respect to genotype. Our results indicate that we can expect only a small number of future cases to arise in both the MV and VV genotypes through primary transmission. This is because the infection risk is assumed to have been very low indeed for several years and so efficient primary transmission to these genotypes is now only possible in combination with rather long incubation periods, such that a substantial proportion of those infected would reach the end of their natural life span before succumbing to clinical disease. Larger numbers of future cases are possible in all genotypes if efficient transmission occurs though red-cell transfusion. However, even these numbers are limited by the numbers of individuals and the age-profile of those that receive transfusions [10], [33]. Our results suggest that if a second epidemic does arise, this is likely to evolve over a number of decades. Our best estimate for the annual incidence is low with up to 10 cases occurring annually, although the credibility intervals are wide due to large uncertainties in many of the key parameters governing transmission.

Despite the rather long time-scale of this potential second wave, we did not find any scenarios which led to a self-sustaining epidemic as classified by the basic reproduction number 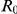. In fact, the 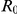 values are so low that even if both leuko-depletion and the donor ban were totally ineffective they would reach values of less than 0.5. This is in contrast with previous work [10], which found the potential for a self-sustaining epidemic for some combination of parameter values in the absence of any control measures. These results were based on fitting to 2 transfusion associated cases up to 2006, whereas here we are fitting to 3 cases up to 2009, taking into account a number of years during which no transfusion associated cases have been observed. Furthermore, some of the assumptions underlying the earlier work were more pessimistic, whereas here we have refined the model to be more realistic, such as allowing for a delay between infection and the onset of infectivity, a lower susceptibility in non-MM genotypes and the use of several red cell units in a single transfusion, reducing the values of the basic reproduction number further.

One assumption implicit in our model simulations concerns the age dependence of susceptibility/exposure to infection. To fit the age distribution of the primary epidemic, as in previous work [27], [28], we fit a strong age dependence in susceptibility/exposure. To date there have been too few secondary cases to fit a distribution to this age profile and we have therefore assumed that for blood-borne transmission susceptibility is independent of age. This might be the case if the age-distribution of cases via primary transmission occurred due to differences in exposure rather than biological susceptibility per se, although evidence for this is limited [34]. Furthermore, animal studies have suggested that one mechanism for biological susceptibility may be age-related changes in the gut [35] and thus it is possible that all ages would be equally susceptible to transmission via blood transfusion. Regardless, even if biological susceptibility did occur via all transmission routes, this remains a reasonable assumption if the infectivity in a single red cell unit is very high. However, for lower transmissibility, including age-dependent susceptibility would reduce the secondary peak considerably given the lack of overlap between those that appear most susceptible to date (teenagers and young adults) and the age distribution of transfusion recipients [25].

We also investigated sensitivity to our assumptions regarding the effectiveness of the control policies in place (see Supplementary Material S1). None of the alternative assumptions investigated changed the overall dynamics significantly, however, the upper limit of the credibility intervals of the projected future epidemic size varied for different scenarios. If leuko-depletion is ineffective in preventing transmissions via red cell transfusions we would expect the secondary outbreak to be up to twice as large, whereas a less effective donor ban had very little effect on the outbreak size. This is because the majority of red cell transfusion cases are caused by people who were themselves infected via the oral transmission route and are therefore not subject to the donor ban. If the test sensitivity of the prevalence test is lower, the true population prevalence is higher than measured in the appendix study, leading to more secondary transmission and therefore potential for a larger secondary outbreak.

In summary, given that there are no further known transmission routes efficient enough that they could lead to a self-sustaining epidemic, the variant CJD epidemic in the UK is likely to continue with a low level annual incidence for a lengthy period of years to decades. Whilst any projections of future case numbers are highly uncertain, reflecting the current uncertainties in key transmission parameters for the genotypes and transmission routes in which we have not seen many cases as yet, the timescales involved are fairly insensitive to these highly uncertain parameters. Despite the inherent large uncertainty our results are important for public health planning: The current low level of annual incidence appears to suggest that the epidemic is nearly over. However, while this might be the case, a secondary peak remains a possibility, and this has to be taken into account when decisions are made about the introduction or withdrawal of control measures.

## Supporting Information

Supplementary Material S1 Details of sensitivity analyses, future transmissions and the basic reproduction number.
(PDF)
