# Supplementary material for: Uncertainty in the Tail of the Variant Creutzfeldt-Jakob Disease Epidemic in the UK
Source: PLoS One. 2010 Dec 23;5(12):e15626. doi: 10.1371/journal.pone.0015626 (PMC3009744; doi:10.1371/journal.pone.0015626)
Supplement: Supplementary Material S1 — Details of sensitivity analyses, future transmissions and the basic reproduction number. (PDF) [file pone.0015626.s001.pdf]

Uncertainty in the Tail of the Variant Creutzfeldt–Jakob Disease Epidemic in the UK – Electronic Supplementary Material

Tini Garske, Azra C. Ghani
MRC Centre of Outbreak Analysis and Modelling, Department of Infectious Disease Epidemiology, Imperial College London, UK

Sensitivity Analyses

Assumptions

In the main paper we have made some assumptions regarding effectiveness of control measures and test sensitivity of the prevalence test. Here, we investigate the sensitivity of our results to these assumptions. Table S 1 shows the four scenarios considered and the associated parameters that differ between them. All other parameters were sampled from the ranges as given in the main text.

Table S 1. Assumptions for the scenarios explored in the sensitivity analyses and number of parameter sets explored.

**Model Parameters**	**Scenarios**			
	Baseline	***Ineffective leuko-depletion***	***Reduced donor ban effectiveness***	***Reduced test sensitivity***
Effectiveness of leukodepletion	40%	0	40%	40%
Effectiveness of donor ban	90%	90%	50%	90%
Test sensitivity	100%	100%	100%	50%
Number of parameter sets explored	1592100	795900	796400	796400

Parameter fitting

The estimated parameters for the different scenarios are shown in Table S 2. Varying the assumptions regarding effectiveness of control measures and test sensitivity has very little impact on these estimates. However, the basic reproduction number R_0 does vary for the different scenarios, indicating the effect of the different control policies. Nevertheless, for none of the scenarios does the upper limit reach anywhere close to the threshold value. Even in the absence of all control measures we would not be in the situation where a self-sustaining epidemic is possible.

Table S 2. Model parameters, prior distributions and medians (95% credibility intervals) of the fitted parameters and R_0 values for the different scenarios.

Parameters			Estimates under different scenarios			
**Description**	**Name**	**Prior distribution**	***Baseline***	***Ineffective leukodepletion***	***Reduced donor ban effectiveness***	***Reduced test sensitivity***
Overall primary infectivity	Ξ_p	U[80,230]	173 (144 – 199)	164 (146 – 195)	170 (146 – 199)	170 (146 – 199)
Mean primary incubation period for MMs	Δ_{MM}	U[10,13.5]	11.6 (10.9 – 12.2)	11.7 (10.9 – 12.2)	11.6 (10.9 – 12.3)	11.6 (11.0 – 12.3)
Incubation period shape parameter	k	U[5,100]	45 (23 – 81)	43 (21 – 76)	41 (23 – 77)	39 (22 – 77)
Age dependent primary susceptibility mean	$\Psi_{p,\text{mean}}$	U[10,40]	17.9 (15.9 – 20.0)	17.9 (16.1 – 20.3)	17.8 (15.7 – 20.4)	17.8 (15.9 – 21.0)
Age dependent primary susceptibility shape	$\Psi_{p,\text{shape}}$	U[1,15]	3.8 (2.3 – 6.6)	3.8 (2.1 – 5.6)	3.8 (2.0 – 7.2)	3.4 (2.0 – 7.2)
Mean primary incubation period for MVs	Δ_{MV}	U[10,80]	34 (19 – 73)	34 (22 – 58)	35 (22 – 72)	35 (24 – 74)
Mean primary incubation period for VVs	Δ_{VV}	U[10,80]	52 (26 – 77)	63 (29 – 77)	54 (29 – 77)	56 (29 – 77)
Basic reproduction number	R_0	n/a	0.0056 (0.0003 – 0.0146)	0.0091 (0.0004 – 0.0227)	0.029 (0.001 – 0.068)	0.0041 (0.0002 – 0.0152)

Epidemic scale

The overall shape and timing of the incidence curve is very similar under the four different scenarios (data not shown), and the point estimates do not differ substantially (see Table S 3). However, the upper limits of the credibility intervals vary somewhat between the different scenarios, with the largest upper limit in the case of inefficient leukodepletion.

For reduced effectiveness of the donor ban, there is very little difference to the baseline scenario. While this might be surprising at first, it is easily explained by the fact that most of the blood transfusion cases are caused by donors that were infected via the primary route and are therefore not subject to the donor ban, whereas those infected via blood transfusions themselves are on average fairly old and do not cause many further transmissions during their remaining lifetime.

Although the test sensitivity is not a control measure, the prevalence in the population between 1995 and 2000 (as measured in the prevalence study) has an impact on the future course of the epidemic, with larger epidemics possible if the prevalence of infection is high, as the prevalent people might transmit the infection to others, whether they will develop clinical symptoms or not. If the sensitivity of the prevalence test is assumed to be only 50%, the true prevalence in the population is expected to be twice as high as the measured prevalence, and therefore the upper limits of the projected future size of the epidemic is considerably higher under this scenario than for the baseline scenario.

Table S 3. Estimated medians (95% credibility intervals) of the cumulative number of future cases from 2010 to 2179 by genotype for the different scenarios.

	Scenario			
	Baseline	***Ineffective leukodepletion***	***Reduced donor ban effectiveness***	***Reduced test sensitivity***
MM	200 (20 – 2200)	140 (28 – 4200)	170 (20 – 2200)	470 (54 – 3000)
MV	160 (4 – 980)	150 (4 – 1600)	140 (5 – 930)	230 (10 – 710)
VV	13 (0 – 85)	12 (0 – 75)	11 (0 – 71)	14 (0 – 150)

Numbers are rounded to two significant digits.

Future transmissions

Due to the long incubation period it is conceivable that there might be a substantial number of cases in the future which have already been infected in the past and would therefore not be preventable with any additional control measures that might be implemented in the future. In order to assess the extent to which infections of future cases have already occurred we performed further simulations for 798500 sets of parameter values in which the transmission rate was set to 0 from 2010 onwards. The total number of future cases is considerably lower than in the baseline scenarios (Table S 4). While the expected number of cases from primary transmission is essentially identical indicating that these transmissions already have occurred, the majority of the transfusion associated cases in the baseline scenario have not been transmitted yet. The time series of the epidemic if no further transmissions occur is shown in Figure S 1, and shows a substantially smaller secondary peak than that of the baseline scenario. The most striking difference to the baseline time series (Figure 6 in the main text) is the sharp cut-off of unidentifiable transfusion associated cases at around 2020 in the growing phase of the epidemic. This means that theoretically, the immediate introduction of effective control measures preventing the transmission via blood transfusions could reduce the number of future cases by around 260 over the next century as a best estimate, or by up to 2600 if the worst case scenarios turn out to be true.

Table S 4. Estimated medians (95% credibility intervals) of the cumulative number of future cases from 2010 to 2179 by genotype and transmission route, assuming that no further transmissions occur from 2010 onwards.

	Transmission route			
**Genotype**	***All routes***	***Primary***	***Identifiable blood***	***Unidentifiable blood***
All genotypes	130 (23 – 400)	99 (17–230)	3 (0 – 27)	18 (1 – 210)
MM	16 (1 – 180)	1 (0 – 6)	1 (0 – 20)	11(0 – 160)
MV	100 (3 – 230)	92 (3 – 210)	1 (0 – 8)	5 (0 – 52)
VV	6 (0 – 38)	6 (0 – 36)	0 (0 – 1)	0 (0 – 5)

Numbers are rounded to two significant digits.

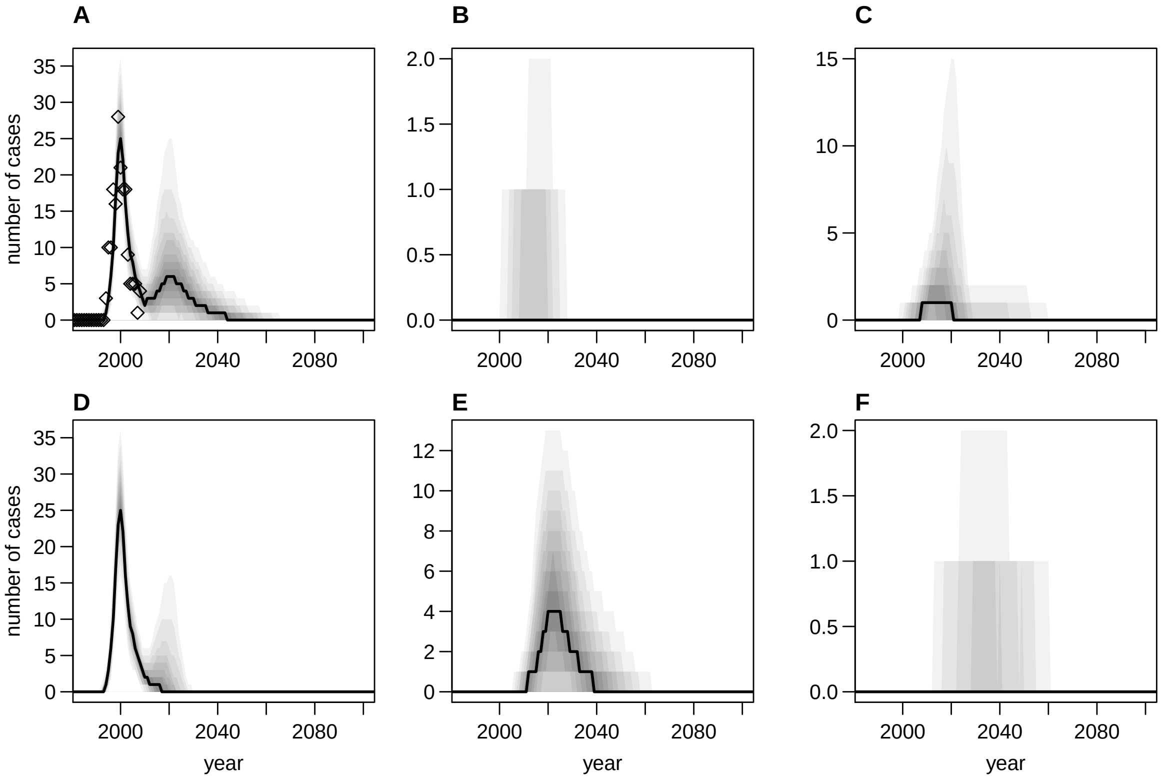

Figure S 1. Median and posterior distributions of projected time series, assuming that no further transmissions occur from 2010 onwards. A Total number of cases, B transfusion associated cases that can be identified through donor-recipient pairing, C unidentifiable transfusion associated cases and D to F number of cases in the different genotypes, MM, MV and VV, respectively. Diamonds = observed epidemic, solid line = median, greyscale graduations: 10% range to 90% range.

The basic reproduction number

The basic reproduction number R_0 is defined as the mean number of onward infections a typical infected individual will cause, and in the limit of an infinite population this has the threshold property that for

any values of $R_0 < 1$, the epidemic will certainly die out, whereas for $R_0 \geq 1$, the epidemic will grow exponentially until saturation is reached unless it dies out through stochastic effects in the early stages. Values of $R_0 \geq 1$ therefore indicate the potential for a self-sustaining epidemic.

In our model the first generation of cases stems from primary infection through the consumption of BSE-contaminated food. Individuals infected via this route are assumed to show different characteristics to those infected in subsequent epidemic generations through human-to-human transmission via red cell blood transfusions, for instance different incubation periods. The concept of the basic reproduction number is valid in the early stages of an epidemic (before any saturation effects become important), but after the stochastic fluctuations of the very first few generations have passed. For calculating R_0, we therefore only need to consider those infected via red cell transfusions.

Formulating the infection process as a multitype branching process, where the type i of any infected individual is determined by their genotype and age at infection, the basic reproduction number R_0 is given as the leading eigenvalue of the next generation matrix $M = (m_{ij})$, where m_{ij} is the expected number of infections of type j caused by a type-i individual [1]. The infection process can be subdivided into two stages, (i) blood donation and (ii) infection following the transfusion.

Neglecting the effects of control measures such as leuko-depletion and the donor ban, individuals donate infectious blood units according to a time-inhomogeneous Poisson process, where the donation rate depends on age and the time since infection, as this determines whether infectivity in the blood has developed yet. The generating function of this Poisson process is given by

$$f^{a,g}_{\text{Poisson}}(s) = \exp\left[n_b(a,g)(s-1)\right] , \tag{1}$$

where $n_b(a,g)$ is the expected number of infectious red cell units donated by a person of genotype g infected at age a during their lifetime, which we shall calculate later. The probability that any infectious red cell unit is transfused into a patient of age a is given by

$$P_b(a) = \frac{\Psi_b(a)b(a)}{\sum_{a'} \Psi_b(a')b(a')} , \tag{2}$$

where $\Psi_b(a)$ is the age distribution of red cell transfusion recipients and $b(a)$ is the mean number of units given in a transfusion to a person of age a. If the unit is transfused to a patient of genotype g, it will cause a new infection with probability $\beta\xi_g$, such that the infection process can be described by a multi-type Bernoulli process with generating function

$$f(\mathbf{s}) = 1 - \beta \sum_g \pi_g \xi_g \left(1 - \sum_a P_b(a) s_{a,g}\right) , \tag{3}$$

where $\mathbf{s} = (s_{a,g})_{a\in\{0,\dots,A\},g\in\{MM,MV,VV\}}$.

The total number of further infections of age a' and genotype g' caused by a person of genotype g who was infected at age a is given as the sum of X iid random variables, where X is the number of infectious blood units donated. The generating function of the two-step process is therefore

$$\begin{aligned} f^{a,g}(\mathbf{s}) &= f^{a,g}_{\text{Poisson}}\left(f_{\text{Bernoulli}}(\mathbf{s})\right) \\ &= \exp\left[-\beta\, n_b(a,g) \sum_{g'} \pi_{g'} \xi_{g'} \left(1 - \sum_{a'} P_b(a') s_{a',g'}\right)\right] . \end{aligned} \tag{4}$$

The expected number of further transmissions of age a' and genotype g' caused by a person of genotype g infected at age a is given by the partial derivative of $f^{a,g}(\mathbf{s})$ with respect to $s_{a',g'}$, evaluated at $\mathbf{s} = \mathbf{1}$,

that is the vector with all entries 1:

$$m_{a,g:a',g'} = \frac{\partial}{\partial s_{a',g'}} f^{a,g}(\mathbf{1}) \\ = \beta n_b(a,g)\pi_{g'}\xi_{g'}P_b(a') \,. \tag{5}$$

As the next generation matrix $M = (m_{a,g:a',g'})$ factorises, in the absence of control measures the basic reproduction number R_0 as its leading eigenvalue can easily be derived as

$$R_0 = \beta \sum_{a,g} \pi_g \xi_g P_b(a) n_b(a,g) \,. \tag{6}$$

If the donor ban has effectiveness e_b, it works by excluding a proportion e_b of those who have ever received a blood transfusion from donating blood, and therefore excluding a proportion e_b of those who have been infected by the blood-borne transmission route from donating. This means that the expected number of units donated by an average infective is reduced to $(1-e_b)n_b(a,g)$, therefore linearly reducing the value of R_0 by this factor. Similarly, the effect of leukodepletion is to reduce the transmission probability β to $(1-e_l)\beta$, where e_l is the effectiveness of leuko-depletion, reducing R_0 by the factor $(1-e_l)$, such that with leuko-depletion and the donor ban in place, the basic reproduction number is given by

$$R_0 = (1-e_l)(1-e_b)\beta \sum_{a,g} \pi_g \xi_g P_b(a) n_b(a,g) \,. \tag{7}$$

The expected number of infectious red cell units donated

For simplicity, the population is structured into age cohorts, with the whole age cohort born at the start of the calendar year, rather than throughout the year. This means that the total population size fluctuates slightly over the year. We have $N(t) = N\exp(-M\phi(t))$, where $\phi(t) = t - \lfloor t \rfloor$ is the phase of the time. At the end of the calendar year, the population has fallen by the number of births into each cohort N_0, and therefore $M = \ln \frac{N}{N-N_0}$. In the British population, the overall annual death rate is $M \approx 1.3\%$pa.

In a population that has a constant birth rate and constant (albeit age-dependent) death rates, the age-composition of the population is stationary over time. At the start of the calendar year, the number of people of age a is proportional to the survival probability to that age, $N_a = N\frac{S(a)}{s}$, where $s = \sum_{a=0}^{A} S(a)$. At any time t, the number of people aged a is $N_a(t) = N_a \exp\left[-\mu_a \phi(t)\right]$, where μ_a is the death rate at age a, which is given by $\mu_a = -\ln S(a+1|a) = -\ln \frac{S(a+1)}{S(a)}$.

We assume that all age-dependent rates (death, donation etc.) are constant per year, so that they don't depend on the exact age a_c, but only on the count of years $a = \lfloor a_c \rfloor$. However, as infection can occur at any exact age, not just at the birthday, for evaluating the number of secondary infections we need to take into account the actual age at primary infection, as this determines when the rates change.

The expected number of infectious blood units donated by a person of genotype g who was infected at age $a_c = a + t_0$, where a is an integer and $t_0 \in [0;1[$, throughout their lifetime is given as

$$n_b(a_c g) = \int_0^\infty \alpha(t|a_c g) P(t|a_c g)dt \,, \tag{8}$$

where $\alpha(t|a_c g)$ is the rate of donation of infectious blood units by a person aged $a_c + t$, given infection at age a_c, and $P(t|a_c g)$ is the probability that the person is alive at $t_0 + t$, given they were infected at t_0 when aged a_c.

Assuming that blood donations match the demand for transfusions,

$$\begin{aligned}\alpha(t|a_c g) &= N(t_0+t)\tau_b \frac{\Phi_b(a_c+t)}{N_{a_c+t}}\, G_{\min g}(t) \sum_{a'} \Psi_b(a')b(a') \\ &= N\exp\left[-M\phi(t_0+t)\right]\tau_b \frac{\Phi_b(a+\lfloor t_0+t\rfloor)}{N_{a+\lfloor t_0+t\rfloor}\exp\left(-\mu_{a+\lfloor t_0+t\rfloor}\phi(t_0+t)\right)}\, G_{\min g}(t)\sum_{a'}\Psi_b(a')b(a') \\ &= s\tau_b \exp\left[(\mu_{a+\lfloor t_0+t\rfloor}-M)\phi(t_0+t)\right]\frac{\Phi_b(a+\lfloor t_0+t\rfloor)}{S(a+\lfloor t_0+t\rfloor)}\, G_{\min g}(t)\sum_{a'}\Psi_b(a')b(a')\,, \end{aligned} \tag{9}$$

where $G_{\min g}(t) = G(t|\kappa_{\min}, \eta_g)$ is the cumulative distribution function of the γ-distributed time to becoming infectious, giving the probability that the person has already entered the infectious stage at time t after infection. It is given as

$$G(t|\kappa,\eta) = 1 - \exp(-\eta t)\sum_{k=0}^{\kappa-1}\frac{(\eta t)^k}{k!}\,, \tag{10}$$

and we assume here that only the scale parameter η depends on the genotype. The mean of this distribution is given by $\Delta = \kappa/\eta$.

As the time-dependence of the rate of donations (for constant age) over the year is an artifact of the separation of the population into birth cohorts, we average the exponential factor over the year and get

$$\alpha(t|a_c g) = s\tau_b \frac{e^{\mu_{a+\lfloor t_0+t\rfloor}-M}-1}{\mu_{a+\lfloor t_0+t\rfloor}-M}\,\frac{\Phi_b(a+\lfloor t_0+t\rfloor)}{S(a+\lfloor t_0+t\rfloor)}\, G_{\min g}(t)\sum_{a'}\Psi_b(a')b(a')\,. \tag{11}$$

The probability of being alive at time t after infection at age a_c is given by

$$\begin{aligned}P(t|a_c g) &= \left[\omega_2 + (1-\omega_2)(1-G_g(t))\right] S(a_c+t|a_c) \\ &= \left[\omega_2+(1-\omega_2)Z_g(t)\right]\frac{S(a+\lfloor t_0+t\rfloor)}{S(a)}\exp\left[\mu_a t_0\right]\exp\left[-\mu_{a+\lfloor t_0+t\rfloor}\phi(t_0+t)\right]\,, \end{aligned} \tag{12}$$

where ω_2 is the probability of sub-clinical infection, and $G_g(t)$ is the cumulative distribution function of the incubation period (10), which is γ-distributed with the same shape parameter η_g, but a different scale parameter $\kappa \geq \kappa_{\min}$ than the latent period. The first factor takes into account potential death from disease for pre-clinically infected individuals, whereas the remaining term accounts for death from other causes. Here, we have introduced the shorthand $Z_g(t) = 1 - G_g(t)$.

The expected number of infectious red cell units donated throughout their lifetime by a person infected when age $a_c = a + t_0$ is given by

$$\begin{aligned}n_b(a_c g) &= \int_0^\infty \alpha(t|a_c g)P(t|a_c g)dt \\ &= \frac{N}{N_a}\tau_b \sum_{a'}\Psi_b(a')b(a')\int_0^{A-a+1-t_0}(1-Z_{\min g}(t))\left[\omega_2+(1-\omega_2)Z_g(t)\right]\Phi_b(a+\lfloor t_0+t\rfloor) \\ &\qquad \cdot \frac{e^{\mu_{a+\lfloor t_0+t\rfloor}-M}-1}{\mu_{a+\lfloor t_0+t\rfloor}-M}e^{\mu_a t_0}e^{-\mu_{a+\lfloor t_0+t\rfloor}\phi(t_0+t)}dt\,, \end{aligned} \tag{13}$$

whereas the expected number of infectious red cell units donated by a person infected at some point while

they were aged between a and $a+1$ is

$$\begin{aligned} n_b(ag) &= \int_0^1 n_b(a_c) dt_0 \\ &= \frac{N}{N_a} \tau_b \sum_{a'} \Psi_b(a') b(a') \int_0^{A+1-a} (1 - Z_{\min g}(t)) \left[\omega_2 + (1-\omega_2) Z_g(t)\right] \\ &\quad \cdot \int_0^1 \Phi_b(a + \lfloor t_0 + t \rfloor) \frac{e^{\mu_{a+\lfloor t_0+t \rfloor} - M} - 1}{\mu_{a+\lfloor t_0+t \rfloor} - M} e^{\mu_a t_0} e^{-\mu_{a+\lfloor t_0+t \rfloor} \phi(t_0+t)} dt_0 \, dt \,. \end{aligned} \tag{14}$$

Here, we set $\Phi_b(a > A) = 0$ (note a is an integer, and therefore this relates to $a_c \geq A+1$) in order to ensure that no transmission can occur after reaching the largest possible age. In order to split up the integral over t_0, we write $u = \lfloor t \rfloor$, with

$$\lfloor t_0 + t \rfloor = \begin{cases} u & 0 \leq t_0 < 1 - \phi(t) \\ u+1 & 1 - \phi(t) < t_0 < 1 \end{cases} \tag{15}$$

$$\phi(t_0 + t) = \begin{cases} t_0 + t - u & 0 \leq t_0 < 1 - \phi(t) \\ t_0 + t - (u+1) & 1 - \phi(t) < t_0 < 1 \,. \end{cases} \tag{16}$$

We therefore have

$$\begin{aligned} \int_0^1 & \Phi_b(a + \lfloor t_0 + t \rfloor) \frac{e^{\mu_{a+\lfloor t_0+t \rfloor} - M} - 1}{\mu_{a+\lfloor t_0+t \rfloor} - M} e^{\mu_a t_0} e^{-\mu_{a+\lfloor t_0+t \rfloor} \phi(t_0+t)} dt_0 \\ =& \Phi_b(a+u) \frac{e^{\mu_{a+u} - M} - 1}{\mu_{a+u} - M} e^{-\mu_{a+u}(t-u)} \int_0^{1-\phi(t)} e^{(\mu_a - \mu_{a+u}) t_0} dt_0 \\ &+ \Phi_b(a+u+1) \frac{e^{\mu_{a+u+1} - M} - 1}{\mu_{a+u+1} - M} e^{-\mu_{a+u+1}(t-(u+1))} \int_{1-\phi(t)}^1 e^{(\mu_a - \mu_{a+u+1}) t_0} dt_0 \\ =& \Phi_b(a+u) \frac{e^{\mu_{a+u} - M} - 1}{\mu_{a+u} - M} e^{-\mu_{a+u}(t-u)} \begin{cases} u+1-t & \mu_a = \mu_{a+u} \\ \frac{e^{(\mu_a - \mu_{a+u})(u+1-t)} - 1}{\mu_a - \mu_{a+u}} & \text{else} \end{cases} \\ &+ \Phi_b(a+u+1) \frac{e^{\mu_{a+u+1} - M} - 1}{\mu_{a+u+1} - M} e^{-\mu_{a+u+1}(t-(u+1))} \\ &\quad \cdot \begin{cases} t-u & \mu_a = \mu_{a+u+1} \\ \frac{e^{\mu_a - \mu_{a+u+1}} - e^{(\mu_a - \mu_{a+u+1})(u+1-t)}}{\mu_a - \mu_{a+u+1}} & \text{else} \end{cases} \end{aligned} \tag{17}$$

Inserting this back we get

$$
\begin{aligned}
n_b(ag) =& \frac{N}{N_a}\tau_b \sum_{a'} \Psi_b(a')b(a') \\
& \sum_{u=0}^{A-a} \Bigg[\Phi_b(a+u)\frac{e^{\mu_{a+u}-M}-1}{\mu_{a+u}-M} \\
& \qquad \cdot \int_u^{u+1} dt(1-Z_{\min g}(t))\left[\omega_2+(1-\omega_2)Z_g(t)\right] e^{\mu_{a+u}(u-t)} \\
& \qquad\qquad \cdot \left\{ \begin{array}{ll} u+1-t & \mu_{a+u}=\mu_a \\ \frac{e^{(\mu_a-\mu_{a+u})(u+1-t)}-1}{\mu_a-\mu_{a+u}} & \mu_{a+u}\neq\mu_a \end{array} \right\} \\
& + \Phi_b(a+u+1)\frac{e^{\mu_{a+u+1}-M}-1}{\mu_{a+u+1}-M} \\
& \qquad \cdot \int_u^{u+1} dt(1-Z_{\min g}(t))\left[\omega_2+(1-\omega_2)Z_g(t)\right] e^{\mu_{a+u+1}(u+1-t)} \\
& \qquad\qquad \cdot \left\{ \begin{array}{ll} t-u & \mu_{a+u+1}=\mu_a \\ \frac{e^{\mu_a-\mu_{a+u+1}}-e^{(\mu_a-\mu_{a+u+1})(u+1-t)}}{\mu_a-\mu_{a+u+1}} & \mu_{a+u+1}\neq\mu_a \end{array} \right\} \Bigg] \\
=& \frac{N}{N_a}\tau_b \sum_{a'} \Psi_b(a')b(a') \sum_{u=0}^{A-a} \left[H_b(a+u)I_-(ag,u)+H_b(a+u+1)I_+(ag,u)\right] , \qquad (18)
\end{aligned}
$$

where

$$
H_b(u) = \Phi_b(u)\frac{e^{\mu_u-M}-1}{\mu_u-M} \qquad (19)
$$

and the integrals are $I_-(ag,u)$ and $I_+(ag,u)$.

Using the definition of $Z_g(t)$ and $G_g(t)$, $I_\pm(ag,u)$ can be rewritten as the sum of integrals of the form

$$
K_m(\theta,u) := \int_u^{u+1} t^m e^{-\theta t} dt \qquad (20)
$$

with $u \geq 0$. Depending on the values of θ and m, this evaluates to

$$
K_m(\theta=0,u) = \frac{(u+1)^{m+1}-u^{m+1}}{m+1} \qquad (21)
$$

$$
K_0(\theta\neq 0,u) = \frac{1}{\theta}\left(e^{-\theta u}-e^{-\theta(u+1)}\right) \qquad (22)
$$

$$
K_{m>0}(\theta\neq 0,u) = \frac{1}{\theta}\left(u^m e^{-\theta u}-(u+1)^m e^{-\theta(u+1)}+mK_{m-1}(\theta,u)\right) \qquad (23)
$$

Defining

$$
\begin{aligned}
L_g(\theta,u) :=& \int_u^{u+1} e^{\theta(u-t)}(1-Z_{\min g}(t))\left[\omega_g+(1-\omega_g)Z_g(t)\right] dt \\
=& e^{\theta u}\Bigg\{\omega_g\left[K_0(\theta,u)-\sum_{m=0}^{\kappa_{\min}-1}\frac{\eta_g^m}{m!}K_m(\theta+\eta_g,u)\right] \\
& +(1-\omega_g)\left[\sum_{m=0}^{\kappa-1}\frac{\eta_g^m}{m!}K_m(\theta+\eta_g,u)-\sum_{m,n=0}^{\kappa_{\min}-1,\ \kappa-1}\frac{\eta_g^{m+n}}{m!n!}K_{m+n}(\theta+2\eta_g,u)\right]\Bigg\} , \qquad (24)
\end{aligned}
$$

and

$$L_{gt}(\theta,u) := \int_u^{u+1} te^{\theta(u-t)}(1 - Z_{\min g}(t))\,[\omega_g + (1-\omega_g)Z_g(t)]\,dt$$

$$= e^{\theta u}\left\{\omega_g\left[K_1(\theta,u) - \sum_{m=0}^{\kappa_{\min}-1}\frac{\eta_g^m}{m!}K_{m+1}(\theta+\eta_g,u)\right]\right.$$

$$\left.+(1-\omega_g)\left[\sum_{m=0}^{\kappa-1}\frac{\eta_g^m}{m!}K_{m+1}(\theta+\eta_g,u) - \sum_{m,n=0}^{\kappa_{\min}-1\ \kappa-1}\frac{\eta_g^{m+n}}{m!n!}K_{m+n+1}(\theta+2\eta_g,u)\right]\right\}, \tag{25}$$

we have

$$I_-(ag,u) = \begin{cases}(u+1)L_g(\mu_a,u) - L_{gt}(\mu_a,u) & \mu_{a+u} = \mu_a\\ \frac{1}{\mu_a-\mu_{a+u}}\left[e^{\mu_a-\mu_{a+u}}L_g(\mu_a,u) - L_g(\mu_{a+u},u)\right] & \mu_{a+u} \neq \mu_a\end{cases} \tag{26}$$

and

$$I_+(ag,u) = \begin{cases}e^{\mu_a}\left[L_{gt}(\mu_a,u) - uL(\mu_a,u)\right] & \mu_{a+u+1} = \mu_a\\ \frac{e^{\mu_a}}{\mu_a-\mu_{a+u+1}}\left[L_g(\mu_{a+u+1},u) - L_g(\mu_a,u)\right] & \mu_{a+u+1} \neq \mu_a\,.\end{cases} \tag{27}$$

References

1. Jagers P (1975) Branching processes with biological applications. Wiley.
